# Preparation and Characterization of Novel Poly(Lactic Acid) Composites Reinforced with “Latxa” Sheep Wool Fibers: The Effect of Peroxide Surface Treatments and Fiber Content

**DOI:** 10.3390/ma17194912

**Published:** 2024-10-08

**Authors:** Aitor Arbelaiz, Telmo Yurramendi, Ander Larruscain, Ane Arrizabalaga, Arantxa Eceiza, Cristina Peña-Rodriguez

**Affiliations:** ‘Materials + Technologies’ Group (GMT), Chemical & Environmental Engineering Department, Faculty of Engineering, Gipuzkoa, University of the Basque Country UPV/EHU, 20018 Donostia-San Sebastian, Spain; telmo.yurramendi@ehu.eus (T.Y.); ander.larruscain@ehu.eus (A.L.); ane.arrizabalaga.aa@gmail.com (A.A.); arantxa.eceiza@ehu.eus (A.E.)

**Keywords:** wool fiber, poly(lactic acid), composite, mechanical properties, fire resistance

## Abstract

“Latxa” sheep wool is rough, and it is not used in the textile industry because the fiber diameter is high compared with other wool fibers. Nowadays, this wool is considered as disposal and, with the aim to give it value, new uses must be explored. In the current work, the “Latxa” sheep wool fiber was evaluated as poly(lactic acid) (PLA) polymer reinforcement. With the objective to optimize fiber/matrix adhesion, fibers were surface modified with peroxide. Oxidation treatment with peroxide led to chemical modifications of the wool fibers that improved the fiber/PLA adhesion, but the strength values achieved for the composites were lower compared to the neat PLA ones. The mechanical properties obtained in the current work were compared with the literature data of the PLA composites reinforced with vegetable fibers. The wool fibers showed inferior mechanical properties compared to the vegetable fiber counterparts. However, the preliminary results indicated that the incorporation of wool fibers to PLA reduced the flammability of composites.

## 1. Introduction

The Latxa sheep breed, originally from Basque Country and Navarra, in the northeast of Spain, is used to produce milk, from which different dairy products are produced such as Idiazabal Cheese, among others. After the sheep are sheared, about 2000 tons of wool are obtained per year, being this wool considered as disposable. Latxa sheep wool cannot be used in textile because it is rough and the fiber diameter is very high compared with other wool fibers used in textile industry [1,2], and consequently other uses must discovered to add value to “Latxa” sheep wool. The value of the wool depends on fiber coarseness, because fibers with 35 µm diameter or higher hardly have applications [3]. In the literature, there are reported works where wool fibers are used for the preparation of biocomposites [4,5,6,7,8,9,10,11,12,13,14,15]. Wool fiber shows interesting mechanical properties and can be used as reinforcement in composites. For example, Alzeer and MacKenzie [4] incorporated long wool fibers into a geopolymer matrix. One limitation of geopolymer when used in the construction and building sectors is its brittle failure mode under applied force. After the incorporation of long wool fibers the prepared composites showed, in addition to an improvement in flexural strength, around a 40% more stable fracture mode. Fiore et al. [5] prepared and studied cement mortar reinforced with wool fibers. They concluded that after the incorporation of wool fibers into mortars, the thermal insulation properties were enhanced but the compressive strength decreased. Salama et al. [6] prepared and characterized polymer composites based on polypropylene and recycled wool micro-powder. They concluded that the composite based on polypropylene (PP) and wool powder showed improved properties. Gama et al. [7] prepared composites using polyurethane residues and textile fiber residues, among them being wool fibers. They observed that after the incorporation of textile fiber residues into polyurethane, the modulus value increased considerably, although the strength and deformation at break diminished. They suggested that these composites can find many applications, in construction or automotive sectors, with the advantage of being produced from 100% recycled raw materials. Alkateb et al. [8] used wool as an energy absorber. They fabricated elliptical tubes with composites based on wool woven and epoxy resin. They observed that the wool composite with 30 wt% fiber content absorbed the highest specific energy.

The flammability and low thermomechanical stability of PLA limits the expansion of this polymer in many applications. In a previous work, the thermomechanical stability of PLA was improved by the addition of vegetable fibers in combination with an annealing process [16]. However, the performance against the fire of composites based on polymeric matrices and vegetable fibers is poor, and fire-retardant and intumescent systems should be incorporated [17,18,19,20]. The main drawbacks of most of commercial flame retardants are that the preparation process involves healthy risk and that it is expensive, in addition to not being environmentally sustainable. Furthermore, usually, adding them to the polymeric matrix deteriorated the mechanical properties of the biocomposites [19]. Shumao et al. [17] added ammonium polyphosphate flame retardant to biocomposites of PLA and ramie fibers. They observed that when flame-retardant loading was 10.5 wt%, the strength decreases considerably. They suggested that flame retardant could hinder the adhesion between PLA and ramie fibers. Bocz et al. [18] prepared flax fiber reinforced PLA/Thermoplastic starch (TPS) biocomposites with glycerol phosphate plasticizer. This plasticizer had a flame-retardant effect; however, after the addition of glycerol phosphate plasticizer, the strength value decreased. They suggested that the presence of glycerol phosphate reduced the compatibility between the cellulosic fibers and the biopolymer matrix. Shukor et al. [20] observed that the flexural strength was reduced in kenaf fiber/PLA biocomposites after the addition of ammonium polyphosphate flame retardant. Therefore, it is a challenge to prepare novel biocomposites with good mechanical properties and flame-retardant properties.

In the literature, it was observed that wool fibers have better fire resistance than vegetable ones, and that the presence in wool fiber of sulfur and nitrogen atoms, around 3 and 15 wt%, respectively, leads to a higher fire resistance than vegetable fiber ones [21]. Wool fiber forms intumescent char during the combustion resulting in low heat of combustion and also high limiting oxygen index [22]. Wool showed the limiting oxygen index (LOI) of 25, whereas plant fibers showed values between 18 and 20 [23]. Najmah et al. [9] prepared building blocks based on wool, sulfur, and canola oil. They suggested that the presence of wool gives to the composite a considerable flame resistance as well as the ability to resist higher temperatures. Moreover, after the incorporation of wool, the modulus of elasticity increased compared to the unreinforced counterpart. On the other hand, Kim et al. [10] observed that the fire-retardant behavior was enhanced in the PP/wool fiber composites in comparison with neat PP. Vasina et al.[11] prepared and characterized different polymer/sheep wool composites. They observed that an increase in the wool content in the composites resulted in the enhancement of sound absorption properties due to the higher conversion of acoustic energy into heat. Tusnim eta al. [12] studied the properties of jute and sheep wool fiber-reinforced hybrid polypropylene composites. They concluded that the mechanical properties increased as the fiber loading was increased and that the best results were obtained at 15% fiber loading with jute and wool fiber ratio of 3:1. In the literature, there is one study where authors investigated wool fiber embedded additive manufacturing-based PLA structures for biomedical applications [14]. Even though some recent publications treat the wool fiber-reinforced polymer composites [11,13,15], to the best of our knowledge no study has dealt with PLA/wool fiber biocomposites prepared by injection molding.

In the current work, novel PLA/wool fiber composites prepared by injection molding were characterized. On the other hand, the mechanical results of prepared composites were compared with a literature survey of PLA-based composites reinforced with vegetable fibers. Finally, preliminary flammability results of PLA/wool fiber composites were compared with PLA composites reinforced with vegetable fibers.

## 2. Materials and Methods

### 2.1. Materials

PLA used in the current work was IngeoTM 3051D (Plymouth, MN, USA) purchased by NatureWorks. According to the supplier, the melt flow index is of 6 g/10 min at 210 °C and it has a density of 1.24 g/cm^3^. “Latxa” sheep wool fiber was provided by a local farmer from Urnieta (Gipuzkoa, Spain). The diameter of wool fibers can be higher than 100 µm [2] and the density is around 1.24 g/cm^3^.

### 2.2. Wool Fiber Treatments

The raw fibers were cleaned with a neutral soap in water at the temperature of 55 °C. After drying the cleaned wool fibers, they were dipped in hydrogen peroxide solution with a concentration of 33%. Around 35 g of wool fiber was dipped in 1 L of hydrogen peroxide solution and different treating times were selected, 30 min and 24 h. After the peroxide treatment, fibers were washed with abundant water and finally they were dried.

### 2.3. Compounding and Processing of Materials

PLA and wool fibers were dried in an oven at 100 °C for 12 h. The fiber loading varied in the composites from 5 to 30 wt%. First, dried PLA pellets were molten in a HAAKE Rheomix 600 internal mixer (Thermo Scientific, Karlsruhe, Germany) at 185 °C. Once the polymer was molten, the dried wool fibers were incorporated, and the mixture was processed for 10 min at 80 rpm. The obtained blends were pelletized and dried in an oven prior to process by injection molding technique using a HAAKE Minijet II machine. Injection was carried out at 185 °C applying a pressure of 650 bar. Tensile test specimens (ASTM-D638-10, type V) were obtained.

### 2.4. Characterization Techniques

#### 2.4.1. Fourier Transform Infrared Characterization

Differences in chemical composition between raw wool fiber and treated fibers were observed by Fourier transform infrared spectroscopy (FTIR). FTIR spectra were obtained in a Nexus 670 spectrometer (Nicolet, Markham, ON, Canada) equipped with a MKII Golden Gate accessory (Specac, Orpington, UK). The measurements were taken in the range between 4000 and 650 cm^−1^ with a resolution of 4 cm^−1^.

#### 2.4.2. Thermogravimetric Analysis

Thermogravimetric analysis (TGA) was performed using a TGA/SDTA 851 analyzer (Mettler Toledo, Greifensee, Switzerland). Samples with weights between 5 and 10 mg were heated from 25 to 800 °C at a heating rate of 10 °C/min in nitrogen atmosphere.

#### 2.4.3. Contact Angle Measurements

Contact angle (CA) values of raw wool fibers and treated sisal fibers were measured with OCA 20 (Data Physics Instruments, Filderstadt, Germany) using HPLC water as test liquid. Samples for CA were obtained by compressing short wool fibers in a mold. The water contact angle of a water droplet deposited on the sample surface was measured.

#### 2.4.4. Differential Scanning Calorimetry

The thermal properties of neat PLA and composites with 30 wt.% of wool fiber were determined by differential scanning calorimetry (DSC). A Mettler Toledo DSC 3+ equipment was used and samples (5–10 mg) were heated from room temperature to 170 °C at a scanning rate of 10 °C/min in nitrogen atmosphere.

#### 2.4.5. Tensile Test

To determine the tensile properties of wool fibers, the cross-section of fiber must be determined. Even though wool fibers show irregular cross-section, for simplicity, fibers with cylindrical shape were considered. The cross-section area was calculated measuring the wool fiber diameter by optical microscopy. For each fiber system, tensile tests were performed using 10 mm gauge length and the rate of 1 mm/min. Fifteen wool fibers were tested for tensile properties calculations. On the other hand, for composites, a minimum of five specimens were tested at the rate of 1 mm/min, and tensile strength, modulus, and the deformation at break were calculated.

#### 2.4.6. Scanning Electron Microscopy

SEM micrographs of the fractured surface of composites were performed by JEOL JSM-6400 (Tokyo, Japan) equipment. Fractured surfaces were previously coated with gold using Q150TES metallizer (Lewes, UK).

#### 2.4.7. Vertical Burn Tests

As preliminary results and for comparison purposes, using the tensile specimens, the flammability and self-extinguishing performance of prepared systems were evaluated using vertical burn tests. Composites with 30 wt% fiber content were burned and, also, the neat PLA specimens were tested for comparison purposes. A Bunsen burner WLD-TEC GmbH (Arenshausen, Germany) was used, the spacing between the top of the burner and the lower end of the tensile specimens was set 70 mm. The flame was applied for 10 s and, after removing it, the performance of samples was observed.

## 3. Results

**Figure 1:**
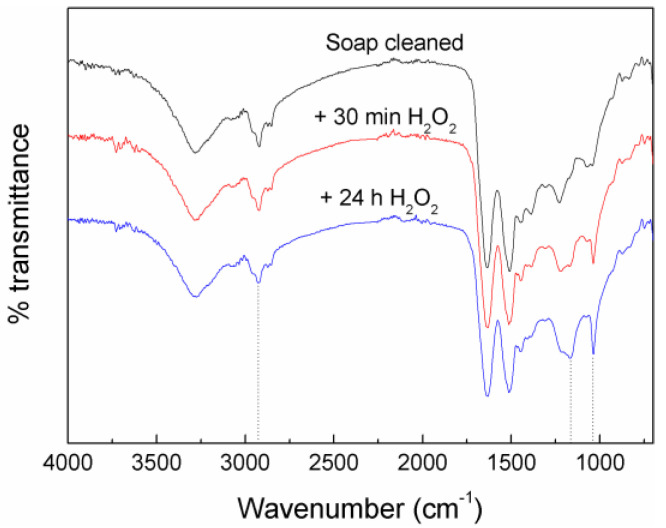
FTIR spectra of studied wool fibers.

Wool fiber is fibrous protein, keratin, with a high abundance of cysteine amino acid. Between protein chains, the cysteine amino acid creates disulphide linkages. These disulphide bonds can be inter- and intramolecular and, consequently, a compact three-dimensional structure is created that stabilizes and insolubilizes the keratin proteins [4,24]. FTIR spectra of different wool fiber systems are shown in Figure 1. Wool fibers showed a strong broad band at 3275 cm^−1^ related to the N-H and O-H stretching vibrations. The bands at 1635, 1508, and 1228 cm^−1^ correspond to amide I, II, and III bands, respectively, related with amino acid groups of wool. The amide I band is attributed to the vibration of C=O groups and the amide II band is related with N-H bending and C-H stretching vibrations. The amide III band is derived from a combination of C-N stretching and N-H bending with contribution from C-C stretching and C=O bending vibrations [25]. The systems treated with peroxide showed a band around 1036 cm^−1^ assigned to the S-O symmetric stretching vibration of cysteine-S-sulphonate or cysteine sulfonic acid [21,26,27,28]. In addition, a new band appeared around 1169 cm^−1^ due to oxidation reactions.

**Figure 2:**
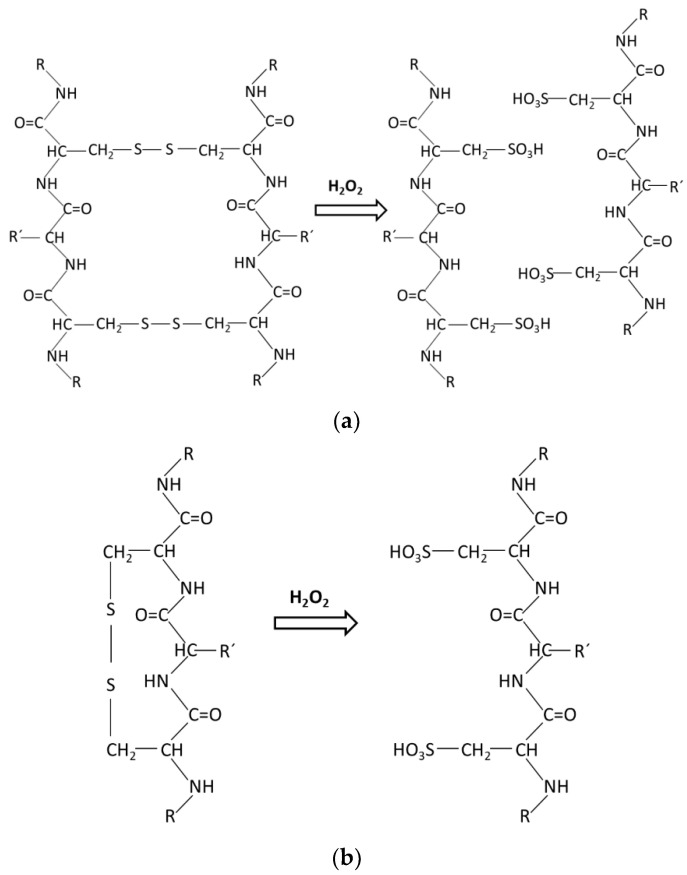
The possible cleavage of intramolecular disulphide bonds due to oxidation treatment with peroxide: (**a**) Intramolecular; scission in two molecules with the surface chemically modified and (**b**) Intramolecular; the surface chemical modification.

In Figure 2, the mechanism of the cleavage reaction of intramolecular disulphide bonds due to oxidation treatment with peroxide was proposed. The intensity of these bands, 1036 and 1169 cm^−1^, increased as the peroxide treatment time was increased. Bhavsar et al. [29] suggested that the variations observed in the region 1000–1300 cm^−1^ are attributed to different sulphur-containing chemical groups of wool that comprise the oxidative disulphide intermediates and the amide III band.

**Figure 3:**
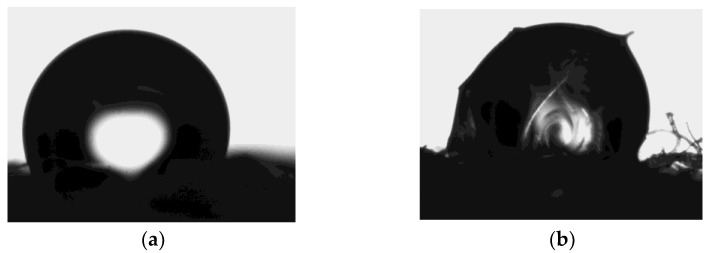
Photographs used for contact angle values measurements: (**a**) soap-cleaned wool fibers and (**b**) peroxide-treated fibers for 24 h.

The outermost layer of a woolen fiber, epicuticle, is made of overlapping scales. These overlapped scales act as liquid water repellent, as can be observed in contact angle photographs of pressed wool fiber disc with a droplet of water (Figure 3). Even though the roughness of the prepared surfaces makes it difficult to determine the contact angle accurately, both systems showed contact angle values between 110 and 120°. Theoretically, the surface for the contact angle should be smooth, but in practice this assumption does not pertain. The lack of smoothness is more evident in the peroxide-treated sample where some fibers can be observed inside the water drop. Gama et al. [7] reported a contact angle of 133° when a drop of water was deposited on the surface of wool.

**Figure 4:**
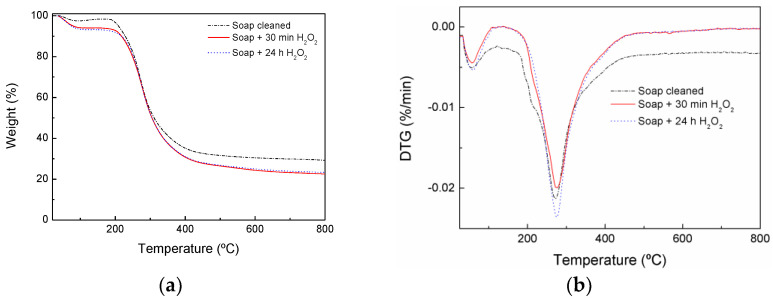
(**a**) Thermogravimetric analysis and (**b**) derivative thermogravimetry curves of wool fibers.

Even though the wool surface acts as liquid water repellent, there are small spaces between the scales from which water vapor can slowly enter the fiber. The TGA curves (Figure 4) showed that all fibers showed around 100 °C, a weight loss related to the water evaporation in agreement with other works [4,21,30]. The weight loss due to water evaporation in peroxide-treated wool systems is higher than for the soap-cleaned counterpart, shown in Table 1. The results suggested that the epicuticle of wool fiber seemed to be damaged by the peroxide treatment and would facilitate the diffusion of water inside of wool fiber. Furthermore, after peroxide treatment cysteine sulfonic acid was created, as observed in FTIR spectra, which led to more possibilities for the creation of hydrogen bonds with water molecules compared to the untreated counterpart, and consequently the water absorption capacity of the treated wool fibers was increased.

The second weight loss is related to the thermal degradation of the wool fibers. The degradation curve of the soap-cleaned wool fiber is slightly different compared to peroxide-treated ones. In the soap-cleaned wool fiber, different small shoulders can be observed at the beginning of this degradation step. Those shoulders could be related with the thermal degradation of low molecular weight compound such as lanolin. In the literature [31,32] it was observed that the lanolin started to degrade around 200 °C and showed a multi-step degradation. Due to that shoulder, the soap-cleaned wool fibers showed a slightly lower onset degradation temperature than the peroxide-treated ones, as shown in Table 1. Wool fiber treated with peroxide for 30 min seemed to show a small shoulder; however, after 24 h treatment this shoulder was missing in the thermogram. The TGA results suggested that after soap cleaning of the fibers, some lanolin is present in the wool fibers, but the peroxide treatment seemed to be effective to remove this residual lanolin. In agreement with the TGA results, the intensity of the absorption band observed by FTIR technique at 2926 cm^−1^, attributed to –CH_2_ stretching [29], seemed to diminish after peroxide treatments (Figure 1). This band reduction could be related with lanolin removal from wool fibers since chemically, lanolin consists of a mixture of several sterols, fatty acids, and their esters [33].

The onset temperatures of the peroxide-treated fibers were superior compared to the soap-cleaned fiber ones, indicating that the peroxide treatment improved the thermal stability of wool fibers. Kim et al. [21] observed that wool fiber started to degrade at around 250 °C due to ruptures of the helical structure, and afterwards cystine disulphide bonds were broken at around 320 °C. At high temperatures, all wool systems showed a significative residue amount, being higher for wool fibers without peroxide treatment, as shown in Table 1. In the literature, the charring ability of wool was observed previously [21].

In Table 2, the tensile properties of wool fibers are reported and compared with literature data of vegetable fibers. The soap-cleaned wool fiber showed strength, modulus, and deformation at the break values of 163 MPa, 6.2 GPa and 16.1%, respectively. It must highlighted that the standard deviation values were high, indicating a high variability in the tensile properties. Mechanical properties differed from one wool fiber to another due to several factors [24]. We noticed that the average diameter values varied after peroxide treatments. The soap-cleaned fiber was around 63 µm, but after the oxidation treatment with peroxide the diameter was reduced to around 50 µm. Based on the proposed cleavage mechanism of Figure 2a, this reaction could be the reason for reducing the fiber diameter. The strength value reported in the current study is in the range of the values reported in the literature [24,34,35]. Zhang et al. [34] reported for merino wool fibers a strength of 151 MPa and an elongation at the break value of 43.5%; unfortunately, they did not report the modulus value. Kim et al. [35] characterized the tensile properties of wool fibers as the average strength, modulus, and strain at the break values of 160.9 MPa, 4.8 GPa, and 27.7%, respectively. Bouagga et al. [24] studied the physico-chemical, thermal, and mechanical properties of Tunisian wool. They reported that wool fibers showed an average tensile breaking force of 16.89 cN, with the average diameter of 28.33 µm. Based on these data, the estimated tensile strength for Tunisian wool is of 268 MPa. The strength data reported in the current study are lower than those estimated for Tunisian wool. Regarding the elongation value, Bouagga et al. [24] reported an average elongation value of 32.5%, being the value reported in the current study lower. However, the young modulus reported by them, 907 MPa, is significantly lower compared to what we determined in the current work.

After the peroxide treatment, the strength value hardly changed. Even though the oxidation reaction with peroxide resulted in chemical modifications of the wool fibers, as observed by FTIR, these modifications did not alter the tensile strength significantly. While the oxidation treatment with peroxide introduced a cleavage of the covalent intramolecular disulphide bonds, it seems that these bonds were not mainly responsible for the strength of the wool fibers [38,39]. Zahn and Blankenburg [40] suggested that the hydrophobic interactions between the chains were responsible for retaining the strength of wool even at high moisture regains. It is probable that the peroxide treatment did not alter hardly the hydrophobic interactions between chains, and consequently the mechanical properties did not alter significantly.

Regarding the modulus, the wool fiber treated with peroxide showed higher modulus values than untreated one. Furthermore, as the treatment time was increased, the modulus value was increased. One possible explanation of this increase would be the removal of lanolin from wool fibers that can act as plasticizer. On the other hand, the modulus increment could be also due to the creation of new H-bonds among different peptide chains thanks to cysteine sulfonic acid groups created in peroxide treatment. As the treatment time was increased, the intensity of FTIR bands related with cysteine sulfonic acid groups was increased, suggesting that the number of cysteine sulfonic acid groups were increased. It should be indicated that the increment observed should be taken with caution due to the high standard deviations.

Regarding the comparison with vegetable fibers, the wool fibers showed higher deformation at the break value compared to the vegetable counterparts. However, the strength and modulus values of vegetable fibers were higher than wool fiber ones, suggesting that the reinforcing effect of wool fibers was, theoretically, lower than the vegetable fibers. The lack of crystallinity of wool fibers [7,21,30], among other reasons, led to a material with lower mechanical properties compared to the vegetable fiber ones. Usually, highly crystalline fibers show higher strength and can increase the stiffness of composites [7].

**Figure 5:**
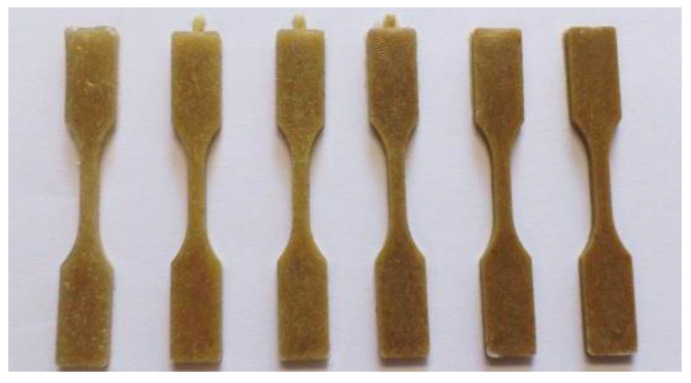
The fiber loading increased from 5 wt.% (**left**) up to 30 wt.% (**right**).

Figure 5 shows the injection molded specimens of PLA/wool fiber with different fiber loading.

**Figure 6:**
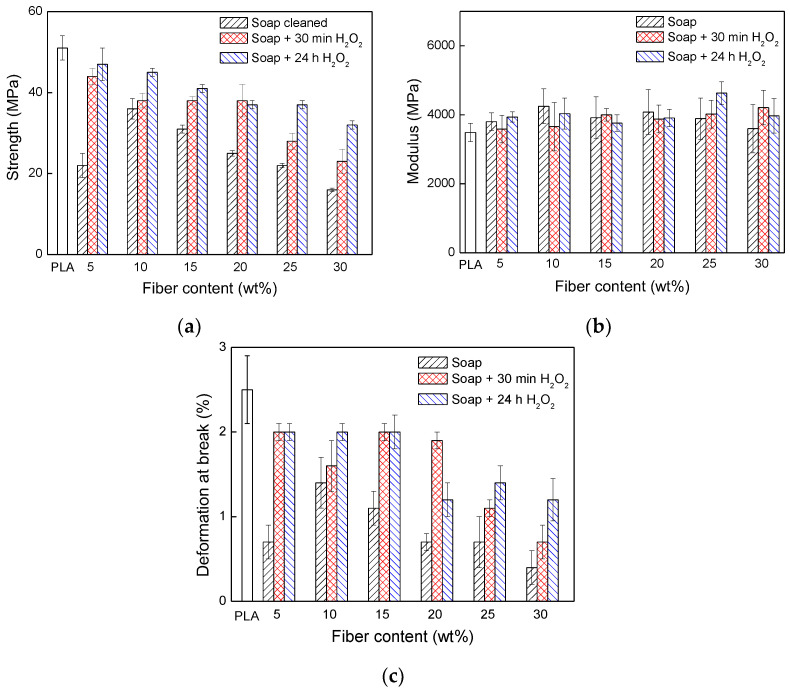
Tensile properties as a function of wool fiber loading and fiber treatment: (**a**) strength; (**b**) modulus and (**c**) deformation at break.

Figure 6 shows the strength values of neat PLA and PLA/wool fiber composites with different fiber loadings. The strength values of the composites were lower than the neat PLA one, indicating that the wool fibers were not reinforcing the polymer matrix. In general, as the content of fiber was increased in the composites, the strength value was decreased. As the strength value of fiber was considerably superior to neat PLA, the results indicated that the wool fiber/PLA adhesion was poor and there was a deficiency of stress transfer from the matrix to the fiber.

The composites that were reinforced with wool fiber washed with soap showed lower strength values than the composites reinforced with the peroxide-treated ones. Probably, the presence of lanolin could, in addition to hampering the fiber/matrix adhesion, reduce the fiber friction during the fiber pull-out. Even though the composites containing the wool fiber treated with peroxide showed superior strength compared to the composites reinforced with wool fiber only washed with soap, the strength values achieved were lower than the neat PLA one. The aspect ratio of the reinforced fiber and the interfacial adhesion ultimately determined the tensile strength of composites. The obtained results indicated that, irrespective to the treatment, the wool fibers were not able to improve the strength in composite due to the poor fiber/matrix adhesion. However, it is clear that the peroxide treatment improved to some extent the fiber/matrix adhesion, but it was not enough. Among the prepared composite systems, the highest strength values were observed in systems treated with peroxide for 24 h. A possible explanation of the strength improvement could be the total removal of lanolin after 24 h in peroxide solution, which is in agreement with TGA results obtained. On the other hand, the peroxide treatment created cysteine sulfonic acid groups that could create new interactions with the PLA matrix, improving the fiber/matrix adhesion. Even though the peroxide treatment improved the fiber/matrix adhesion, the strength values obtained indicated that the created new fiber/matrix interactions were weak. Mangat et al. investigated wool fiber embedded additive manufacturing-based PLA structures [14]. The direct comparison of data of specimens prepared by 3D printed parts and injection molded specimens in the current study had no relevance. Due to the layer-by-layer construction, the 3D printed specimens showed considerably lower mechanical strength compared to the injection molded specimens.

**Figure 7:**
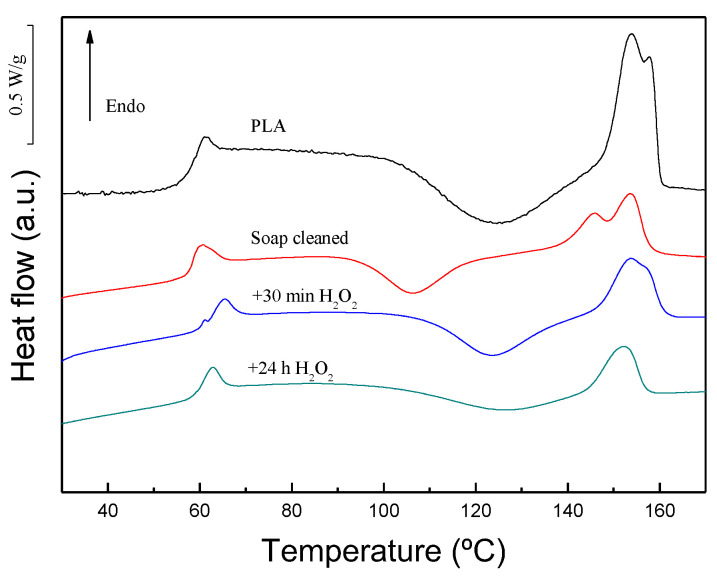
DSC thermograms of neat PLA and composites with 30 wt% of wool fiber with different surface treatments.

Regarding the modulus values, after the incorporation of wool fibers, a slight increase was observed irrespective of the fiber treatment and fiber loading. Neat PLA is a brittle polymer at temperatures below 60 °C, and consequently the value of deformation at break is very low, around 2.5%. After the incorporation of fibers, the deformation at the break values decreased, as the decrease was more marked for composites reinforced with only soap treated fibers. The deformation at the break values indicated that after the peroxide treatment the systems can elongate more, due to fiber/matrix adhesion improvement, and consequently higher strength and deformation at the break values were achieved compared to the composites without peroxide treatment. Gama et al. [7] observed a similar trend after the incorporation of wool fibers to polyurethane matrix. They prepared composites using polyurethane residues and textile fiber residues such as cotton, wool, and polyesters fibers to produce 100% recycled composites. The fiber loading of the composites varied from 50 to 70 wt%. The addition of wool fibers led to a significant decrease in maximum stress from 18 MPa, for neat PU, to values located in the 5–6.5 MPa range. The results obtained by Gama et al. [7] suggested that the wool fibers/PU adhesion was poor, and consequently the wool fibers appeared to be acting more as filler than reinforcement. It should be mentioned that the fiber amount incorporated by Gama et al. [7] is very high and probably some fibers were not well wetted with the PU matrix. On the other hand, they observed that after adding wool fibers the tensile modulus increased greatly. For example, in composites reinforced with 70 wt% of wool fiber, the modulus increased from 135 MPa, for neat PU, to 325 MPa. Xu et al. [30] observed that after adding wool powder to PP the mechanical properties were reduced as the loading of the wool powder was increased in the composite. They blended wool powder with polypropylene via extrusion using glycerol as plasticizer, and the extruded pellets were hot-pressed obtaining a film. They concluded that even though wool powder incorporation to polymer destructs the integrity of PP, no strong linkage was created at the wool/PP interface. Kim et al. [21] compared the mechanical properties as well as the flammability of polypropylene composites reinforced with natural fibers. In contrast to the results observed in the current study, they observed strength improvement after wool fiber was added. When 25 wt% of wool fiber was incorporated to polypropylene matrix (72 wt%) with a coupling agent of maleic anhydride grafted polypropylene (3 wt%), they observed that the tensile strength increased from around 30 MPa, for neat PP, to 34.5 MPa for wool composites. On the other hand, the tensile modulus increased from around 1.5 GPa, for neat PP, to 2.4 MPa for wool composites. The strength and modulus improvements indicated that wool fibers were acting as a reinforcement when they were incorporated to the PP matrix with an adequate coupling agent. Salama et al. [6] prepared polypropylene/recycled wool micro-powder composites with different wool contents varying from 1 to 10%. They observed that after adding wool powder to the PP matrix, the maximum stress value of the composites increased with respect to the neat PP. Probably, the wool scales led to a rough topography that could result in some extent of polymer/wool interaction [6,41]. The SEM micrograph of the fractured surface of wool powder composites showed that the adhesion between the wool powder and polypropylene was good, and they did not observe noticeable aggregations. On the other hand, the authors suggested that this strength improvement was in agreement with crystallinity increase observed by the DSC technique. In the current study, the DSC measurements were carried out, and Figure 7 shows the DSC thermograms of the neat PLA and composites with 30 wt% of wool fiber with different surface treatments. All systems are almost amorphous irrespective of the fiber addition and surface treatment. Consequently, the strength variations cannot be ascribed to crystallinity variations.

The neat PLA shows a cold crystallization process during the heating process and after this, the melting process of crystalline fraction. The temperature range of the cold crystallization process of the neat PLA and for composites with peroxide-treated fibers is similar. However, for composites with the soap-cleaned fibers, the cold crystallization process shifted to considerably lower temperatures. This fact suggested that wool fibers cleaned with soap act as a nucleating agent, although after the peroxide treatment the nucleation sites of the fiber surface seemed to be deactivated. Regarding the crystallinity, all systems are almost amorphous irrespective of the fiber addition and surface treatment. The glass transition temperatures of all systems are similar.

Alzeer and MacKenzie [4] prepared and characterized composites of aluminosilicate inorganic polymer with wool fibers. They observed that the addition of wool fibers increased the flexural strength by about 40% with respect to unreinforced system. They proposed the hydrogen bond formation between the wool fibers and the hydroxyl groups in the aluminosilicate inorganic polymer. On the other hand, Fiore et al. [5] observed that the addition of the wool fibers to cement-based composites leads to a notable drop in the mechanical properties. They suggested that the low interfacial adhesion between the fiber and cement matrix creates defects within the cement.

**Figure 8:**
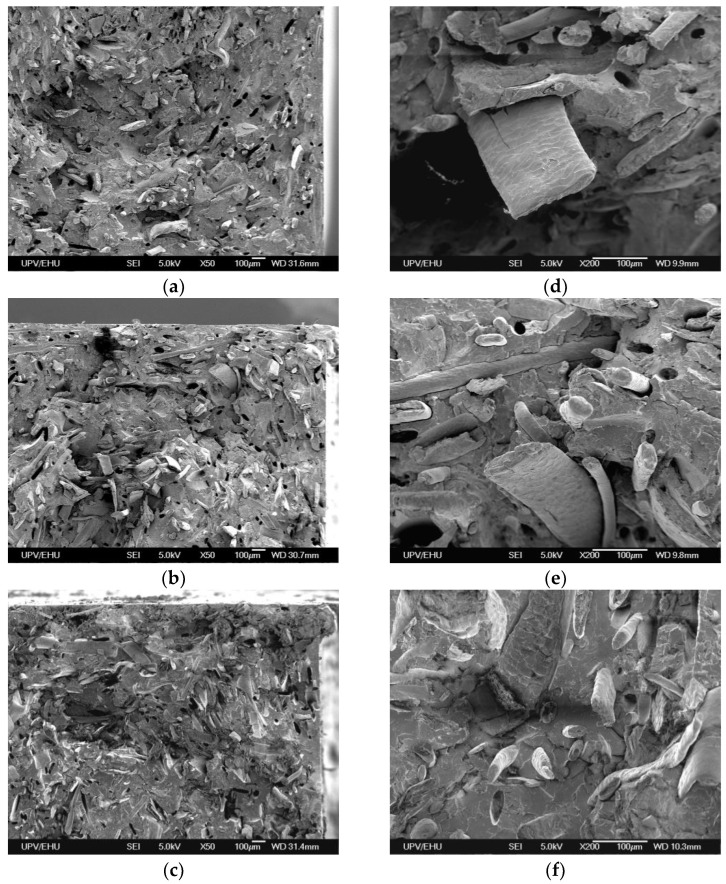
Fractured surface SEM micrographs of wool fiber-reinforced PLA composites with a magnification of ×50: (**a**) soap-cleaned fibers; (**b**) 30 min peroxide-treated fibers and (**c**) 24 h peroxide-treated fibers. A magnification of ×200: (**d**) soap-cleaned fibers; (**e**) 30 min peroxide-treated fibers and (**f**) 24 h peroxide-treated fibers.

The SEM micrographs of the fractured cross-sections of the composites can be observed in Figure 8. In all systems, the dispersion of wool fibers within the PLA matrix is homogeneous. In the SEM micrographs, different multiscale fibers were observed, and the coarseness fibers were around 100 µm in width. In the coarseness fibers, scales could be observed in the cuticle and, for the system treated for 24 h with peroxide, the cuticle imprints of pulled-out fibers on the matrix surface also could be observed.

Regarding the fiber/matrix adhesion, in all systems holes created due to fiber pull-out could be observed as being pulled out fiber surface clean, without matrix residue. These facts corroborate a poor fiber/matrix adhesion, which is in agreement with mechanical results reported previously. However, observing the SEM micrographs with the highest magnification (Figure 8d–f)), it seemed that the interfacial gap between the fiber and the matrix is more obvious in systems reinforced with soap-cleaned and 30 min peroxide-treated fibers, compared to the 24 h peroxide-treated one. The SEM micrographs showed small differences between the studied systems, and the differences observed corroborated the poor fiber/matrix adhesion for all systems, as the fiber/matrix adhesion improved slightly after the peroxide treatment for 24 h.

In Table 3, the tensile properties for the composites with 30 wt% wool fibers obtained in the current study are compared with literature data of the PLA composites reinforced with vegetable fibers. It should be pointed out that the PLA matrix used and the fiber wt.% content for all composites is the same. Composites reinforced with vegetable fibers and without either surface treatment and coupling agent showed similar or slightly higher strength values compared to neat PLA one. When the coupling agent was used, the strength improvement was more pronounced. Regarding the modulus value, vegetable fiber-reinforced composites showed a higher modulus value than wool fiber-reinforced counterparts. From the values reported in Table 3, it can be concluded that the reinforcement capability of vegetable fibers is higher than the wool fiber ones in the PLA-based composites. It is probable that the better wettability of vegetable fibers with molten PLA and the superior tensile properties of vegetable fibers led to composites with better mechanical properties compared to the wool-reinforced counterparts.

**Figure 9:**
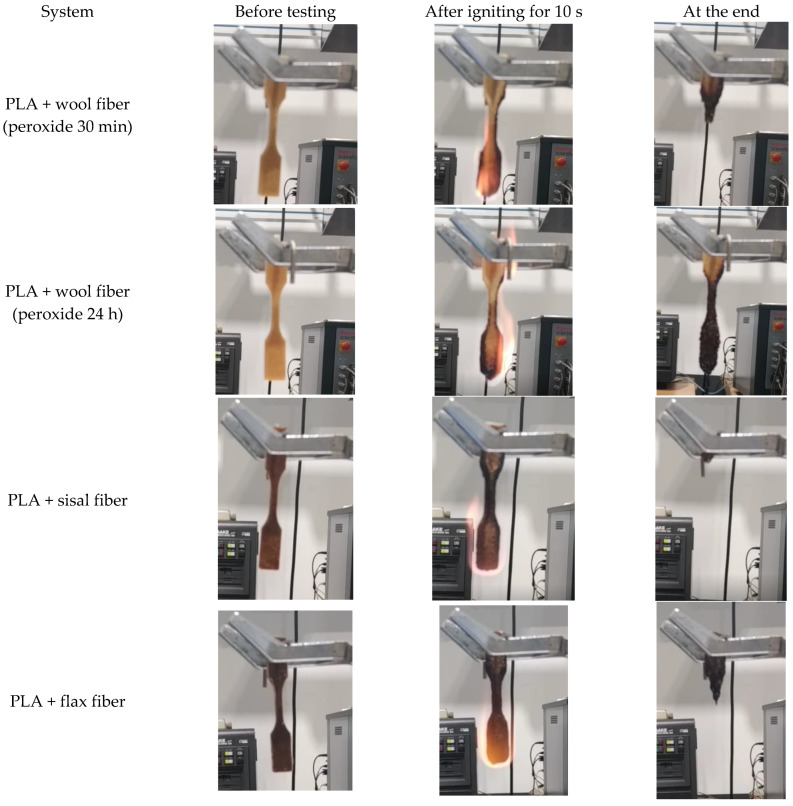
The appearance of PLA/natural fiber specimens with 30 wt.% of fiber loading before, during, and at the end of vertical burn test.

**Figure 10:**
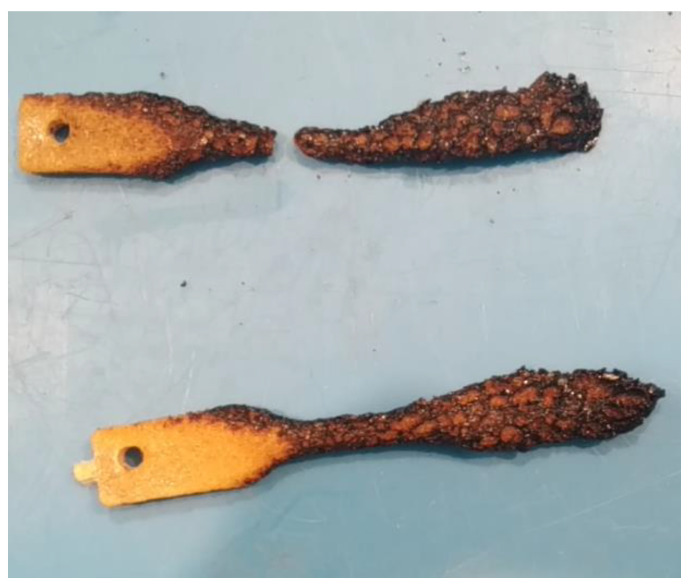
The appearance of specimens after vertical burn test with 30 wt.% of peroxide-treated wool: 30 min peroxide-treated (up) and 24 h peroxide-treated (down).

Preliminary results of the vertical burn test conducted for composites with 30 wt.% of fiber loading are shown in Figure 9. Even though the test is not under any standard, the test compared the flammability of materials. The PLA specimen used is almost amorphous, and consequently the PLA lost the structural integrity at the temperature around 60 °C and started to flow during the flame application. In the vertical burning test, only results of composites with 30 wt.% wool fiber content were included because composites with lower fiber content did not self-extinguish the fire. The incorporation of fibers, both wool and vegetable fibers, improved the structural integrity with respect to unreinforced system since, during the flame application, they did not flow as the neat PLA did. However, all composite systems dripped flaming polymer to a greater or lower extent, irrespective of the fiber type. Obvious differences were observed in fire contact performance between wool-reinforced and vegetable fiber-reinforced systems. The sisal fiber-reinforced composite showed a continuous burning; by contrast, the addition of wool fiber to the polymer gave the composites some flame-retardant properties. For example, composites reinforced with 24 h peroxide-treated wool fibers had self-extinguishment, thus reducing the flammability (see Supplementary Materials video files). The formation of char on the material surface seems to contribute to the suppression of flammability. The char formed seemed to impede the flame penetration and led to extinguish the flame. The char produced during burning seems to act as a protective barrier of the material against the flame and hinders oxygen access to underlying composite (Figure 10). The flame resistant property of wool is due the high moisture and high nitrogen contents of fibers that result in its high ignition temperature [9,43].

## 4. Conclusions

Novel composites based on poly(lactic acid) and “Latxa” sheep wool were prepared and characterized. Results indicated that the oxidation treatment with peroxide led to chemical modifications of wool fibers, as observed by FTIR spectra and TGA thermograms. However, the strength of wool fibers did not alter after peroxide treatment. The chemical modification with peroxide resulted in composites with improved strength values with respect to non-oxidated fiber counterparts. However, the strength values achieved by the composites were lower compared to the neat PLA, indicating that the fiber/matrix adhesion was poor even though fibers were oxidated with peroxide. The SEM micrographs of the fractured surface corroborated that wool fiber/PLA adhesion is poor irrespective of the surface treatment used. The preliminary results indicated that the PLA composites with 30 wt.% of wool fiber self-extinguished the fire. The incorporation of wool fibers to PLA reduced considerably the flammability, but further flammability characterization tests must be performed. These novel composites 100% based on renewable materials could be used in construction or automotive sectors.

## Figures and Tables

**Table 1:** The first mass loss percentages, the onset and maximum degradation temperatures of second mass loss and the char percentages for wool fibers.

Wool Fiber	1st Weigth Loss	2nd Weigth Loss		Char at 800 °C
	(%)	T_onset_ (°C)	T_max_ (°C)	(%)
Soap cleaned	2.5	195.7	272.7	30.5
Soap + H_2_O_2_ 30 min	5.9	203.5	275.6	25.1
Soap + H_2_O_2_ 24 h	6.9	209.3	275.2	24.4

**Table 2:** Tensile strength, modulus, and deformation at break values of different natural fibers.

Natural Fibers	Strength (MPa)	Modulus (GPa)	Deformation at Break (%)	Reference
Soap cleaned	163 ± 23	6.2 ± 2.0	16.1 ± 7.1	Current work
Soap + H_2_O_2_ 30 min	160 ± 33	6.7 ± 2.3	10.0 ± 7.5	Current work
Soap + H_2_O_2_ 24 h	170 ± 33	8.2 ± 3.6	19.0 ± 12.0	Current work
Flax fiber	802	46.9	1.5	[36]
Sisal fiber	366	9.5 ± 3.4	3.9 ± 1.3	[37]

**Table 3:** Comparison of tensile properties of composites based on PLA matrix and different natural fibers.

Systems	Fiber Treatment	Fiber (wt.%)	Coupling Agent	Strength (MPa)	Modulus (GPa)	Reference
Neat PLA	--	0	No	51.0 ± 3.0	3.5 ± 0.3	Current work
PLA/wool fiber	Soap cleaned	30	No	16.0 ± 0.8	3.6 ± 0.7	Current work
PLA/wool fiber	Soap + H_2_O_2_ 30 min	30	No	23.0 ± 3.0	4.2 ± 0.6	Current work
PLA/wool fiber	Soap + H_2_O_2_ 24 h	30	No	32.0 ± 1.2	4.0 ± 0.5	Current work
PLA/flax fiber	No	30	No	52.6 ± 5.8	5.6 ± 0.4	[42]
PLA/sisal fiber	No	30	No	56.5 ± 1.0	5.0 ± 0.3	[42]
PLA/flax fiber	No	30	Yes	67.2 ± 4.5	5.9 ± 0.6	[42]
PLA/sisal fiber	No	30	Yes	62.0 ± 1.8	5.0 ± 0.3	[42]

## Data Availability

The datasets generated during and/or analyzed during the current study are available from the corresponding author on reasonable request.

## Supplementary Materials

The following supporting information can be downloaded at: https://www.mdpi.com/article/10.3390/ma17194912/s1, Video S1: Burning test of neat PLA. Video S2: Composite with wool treated in peroxide 30 min. Video S3: Composite with wool treated in peroxide 24 h. Video S4: Composite with sisal fiber. Video S5: Composite with flax fiber.
